# Analysis of Air Mean Temperature Anomalies by Using Horizontal Visibility Graphs

**DOI:** 10.3390/e23020207

**Published:** 2021-02-08

**Authors:** Javier Gómez-Gómez, Rafael Carmona-Cabezas, Elena Sánchez-López, Eduardo Gutiérrez de Ravé, Francisco José Jiménez-Hornero

**Affiliations:** GEPENA Research Group, University of Cordoba, Gregor Mendel Building (3rd Floor), Campus Rabanales, 14071 Cordoba, Spain; f12carcr@uco.es (R.C.-C.); g02saloe@uco.es (E.S.-L.); eduardo@uco.es (E.G.d.R.); fjhornero@uco.es (F.J.J.-H.)

**Keywords:** complex networks, horizontal visibility graph, time series analysis, mean temperature, topological properties

## Abstract

The last decades have been successively warmer at the Earth’s surface. An increasing interest in climate variability is appearing, and many research works have investigated the main effects on different climate variables. Some of them apply complex networks approaches to explore the spatial relation between distinct grid points or stations. In this work, the authors investigate whether topological properties change over several years. To this aim, we explore the application of the horizontal visibility graph (HVG) approach which maps a time series into a complex network. Data used in this study include a 60-year period of daily mean temperature anomalies in several stations over the Iberian Peninsula (Spain). Average degree, degree distribution exponent, and global clustering coefficient were analyzed. Interestingly, results show that they agree on a lack of significant trends, unlike annual mean values of anomalies, which present a characteristic upward trend. The main conclusions obtained are that complex networks structures and nonlinear features, such as weak correlations, appear not to be affected by rising temperatures derived from global climate conditions. Furthermore, different locations present a similar behavior and the intrinsic nature of these signals seems to be well described by network parameters.

## 1. Introduction

The global increase of surface air temperatures on different time and spatial scales was confirmed in past decades by distinct studies [1,2,3]. Each of the last three decades has been successively warmer at the Earth’s surface than any other preceding decade since 1850 [4]. Some consequences include change in migrations patterns and abundances of many terrestrial, freshwater, and marine species, an increase of vulnerability of some ecosystems and many human systems, shrinking of glaciers, or negative impacts in crops like wheat or maize yield in many regions, influencing current global politics and society [5]. As a result, a major interest in climate variability has appeared among researchers. Most studies have used climate models and statistical approaches to investigate extreme events linked to global warming. The major level of confidence associated with these extreme events are related to extreme heat and cold events [6]. Therefore, the study of temperature variables is a widespread research field [7,8,9,10,11].

In the last decades, a methodology which transforms time series into complex networks was developed [12]. It is called visibility graph (VG) and it has been demonstrated that these graphs inherit the nature of the underlining time series [12,13,14]. Furthermore, this method has been proven to be robust when applied to different environmental signals [15,16,17,18,19]. A simpler version of this approach, the horizontal visibility graph (HVG), was firstly published by Luque et al. in 2009, who developed a theoretical framework for uncorrelated time series which supported the numerical results [20]. In addition, Lacasa and Toral found that a characteristic exponent of a network property, the degree distribution, was a limit which allowed us to distinguish between chaotic and correlated stochastic nature of time series [14]. As an example of application, Braga et al. described annual evolution of river flow fluctuations in Brazil for more of 80 years data series with HVG [21]. They found significant trends in networks properties.

Although graph theory and other particular complex networks techniques have been used in several works for climate studies [22,23,24], they mainly focus on the spatial description by considering stations and/or grid points as nodes in the so-called climate networks. Moreover, some non-trivial assumptions based on different measures of correlation are generally done to determine connections between nodes. On the contrary, VG’s aim is to obtain a graph from each time series with its links following the same visibility criterion. To best of our knowledge, no previous study has applied an HVG approach to investigate the annual evolution of temperature by means of the topological properties of their graphs.

In this work, the authors’ objective is to explore whether a VG framework can be applied to air mean temperature time series and to verify how some topological properties might change in a warming context. To that end, we used the HVG approach on yearly temperature anomalies of a 60-year period. Three relevant parameters have been analyzed, as in the work of Braga et al. for flow fluctuations of Brazilian rivers, namely: average degree centrality, degree distribution exponent, and global clustering coefficient.

This manuscript is organized as follows: Section 2 presents a detailed explanation of data and methodology used. Data, stations information and the preprocessing technique employed are introduced respectively in Section 2.1 and Section 2.2 The HVG algorithm is presented in Section 2.3 and network topological properties can be found on Section 2.3.1 (degree centrality) and Section 2.3.2 (global clustering coefficient). In Section 3, a description and discussion of the main results are shown, organized as degree centrality computation in Section 3.1 and clustering coefficient computation in Section 3.2. Finally, the most important conclusions are stated in Section 4.

## 2. Materials and Methods

### 2.1. Data

**Figure 1:**
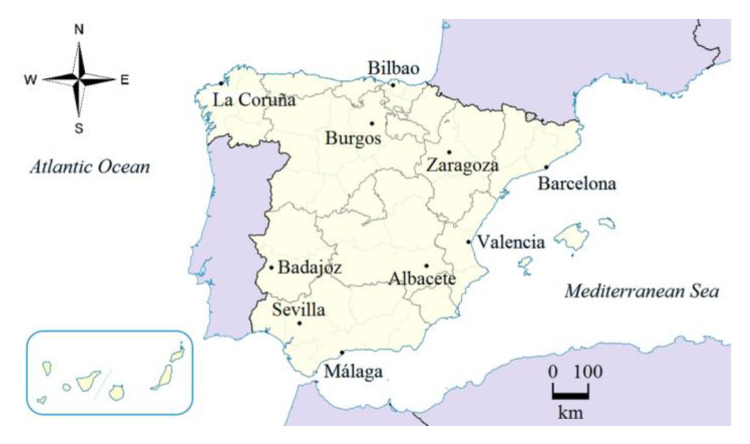
Meteorological stations located over the Iberian Peninsula (Spain) selected for this study.

To undertake this work, daily mean temperature time series from 10 different meteorological stations of Spain located over the Iberian Peninsula were analyzed (see Figure 1). These locations were chosen in order to have the least number of missing data, retaining a representative amount of stations to cover the Atlantic and the Mediterranean semiarid climates, which are the most representative climates in the Iberian Peninsula. In addition, half of them belong to mainland areas and the rest are coastal. In Table 1, we show their names, coordinates, and altitudes. Raw data are publicly available and provided by Agencia Estatal de Meteorología (Spanish Meteorological Agency). The period considered for this study extend to 60 years, from 1960 to 2019.

### 2.2. Seasonality Removal

**Figure 2:**
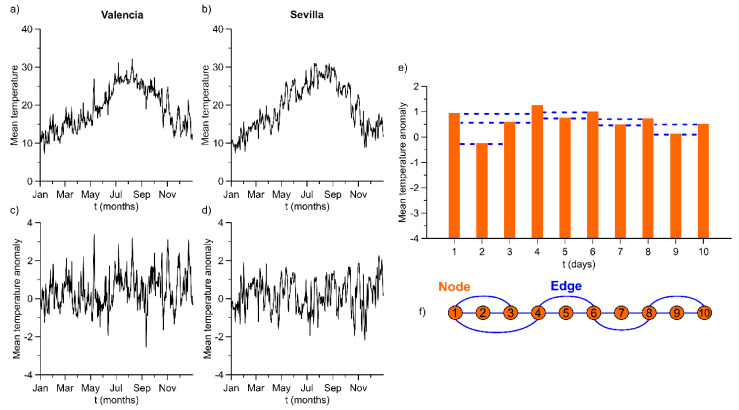
The left margin: (**a**,**b**) Plots that illustrate mean temperature time series of Valencia and Sevilla in the year 2019, respectively. (**c**,**d**) The corresponding anomalies for the same stations and period. The right margin: (**e**) Example of application of the horizontal visibility graph (HVG) algorithm to the first ten values of Sevilla anomalies in 1960. (**f**) Network obtained from the previous plot.

Before employing the HVG algorithm, seasonal behavior of signals has been removed by computing the mean temperature anomalies. These new time series are obtained by subtracting the average value for each calendar day over the whole period from the original time series (μ) and normalizing by their standard error (σ), i.e., $x{’}_{i}=\left(x_{i}-\mu_{i}\right)/\sigma_{i}$, for i=1…366 day [25]. In Figure 2a,b, two examples of time series have been depicted. For illustrative purposes, only one year is shown (2019). Figure 2c,d contain their respective computed anomalies for the same period.

**Figure 3:**
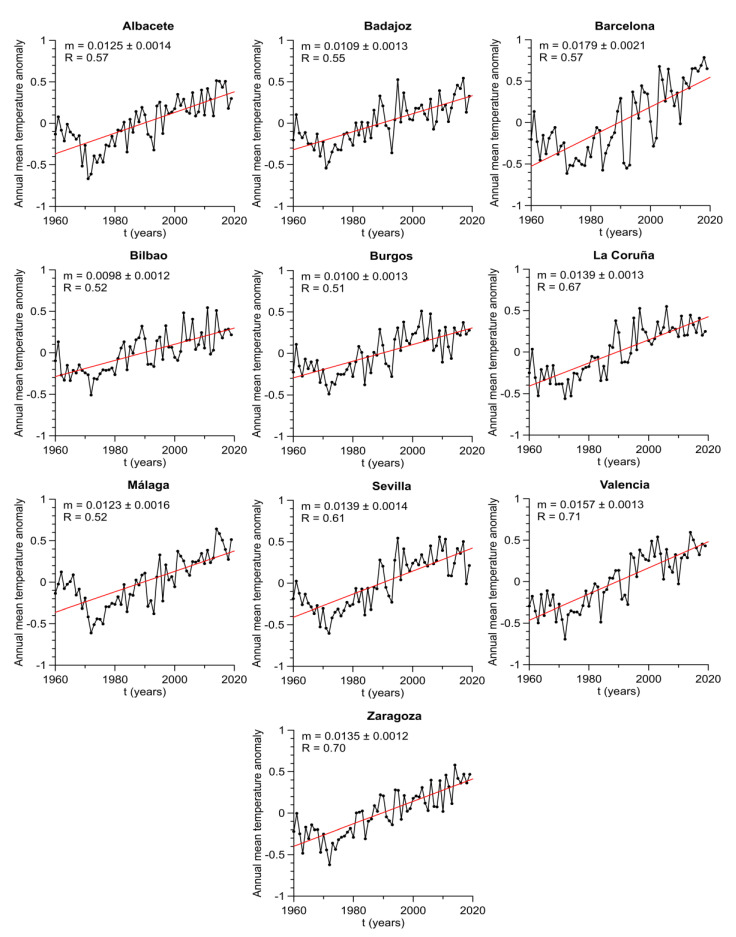
Annual average of daily mean temperature anomalies for every location.

After computing temperature anomalies, one can obtain a better description of annual changes because the seasonal effect has been eliminated. The evolution of annual average values of these anomalies has been explored as a preliminary study. It has been found that all locations show time series with clear upward linear trends (see Figure 3). *t*-tests reject in every case the null hypothesis, which tests whether these slopes are equal to zero; therefore, they are statistically significant. Pearson correlation coefficients are in the range 0.51–0.71 and slopes vary between 0.0098 and 0.0179 °C/year. These trends can be associated to the global climate conditions because they are influenced by the global warming effect.

### 2.3. Horizontal Visibility Graph (HVG)

VGs were conceived by Lacasa et al. in 2008 as an approach that allowed us to transform time series into complex networks [12]. This technique was proven to capture the main nonlinear features of time series such as correlations. One year later, a geometrically simpler procedure of mapping time series was firstly published by Luque et al. with the advantage of being easier to find a theoretical framework that support the recent findings for uncorrelated time series: the horizontal visibility graph (HVG) [20].

The HVG algorithm states that two nodes (or points in a time series) i and j are connected if every node between them fulfills the following criterion:

(1) $$x_{i},x_{j}>x_{p},\;\;\forall \;p\;|\;i<p<j$$

An example of the application of this algorithm can be seen in Figure 2e,f. For more details, some properties of HVGs can be found in Ref. [20].

As every node in HVGs is connected and these connections are bidirectional, the resulting network is connected and undirected. Therefore, an HVG can be easily described by their nodes and links, also named edges. By doing so, a natural way of describing this kind of networks is by means of a matrix where each element $A_{ij}$ is one or zero if nodes i and j are connected or not, respectively. The matrix obtained in this way is a n×n adjacency binary matrix, with n being the size of the time series [16].

Some topological properties from the complex networks have been explored in this work, such as the degree distribution and the global clustering coefficient, which are defined further in the text. The procedure used to obtain the time evolution of these topological properties was: (i) to split each time series into each year; (ii) to transform time series to their respective HVG and (iii) to compute the degree centrality and the global clustering coefficient in each case. After that, we investigated the mean values and trends of both topological properties, as Braga et al. did for river flow fluctuations [21].

#### 2.3.1. Degree Centrality

The first studied topological property is the degree centrality, one of the most widely used in several articles due to the simplicity of its computation and the information which provides about the nonlinear properties of time series [12,18,19,26]. This measure is defined as the number of edges, $k_{i}$, that each node i has in the network, i.e., the number of other nodes which node i sees. By using the adjacency matrix, this quantity is formally defined as:

(2) $$k_{i}=\overset{n}{\underset{j=1}{\sum }}A_{ij}\;\;\forall \;j=1\ldots n$$

After computing the degree of every node, the degree probability distribution, $P\left(k_{i}\right)$, can be obtained. For HVGs, the theoretical degree distribution of a random uncorrelated series was demonstrated in [20] and it fits an exponential function: $P\left(k\right)=\left(1/3\right){\left(2/3\right)}^{k-2}$. This expression can be rewritten as $P\left(k\right)\;\sim \;\exp\left(-\gamma k\right)$ with a characteristic exponent value of $\gamma_{un}=\ln\left(3/2\right)$.

As commented in Section 1, Lacasa and Toral found that this theoretical result is also a quantitative frontier between chaotic and correlated stochastic processes [14]. They showed that chaotic time series map into HVGs whose degree distribution follow an exponential function with a characteristic exponent $\gamma <\ln\left(3/2\right)$ (λ in the original work) whereas correlated stochastic series exhibit exponential degree distributions as well, but with $\gamma >\ln\left(3/2\right)$. Moreover, every possible value on the left and the right of $\gamma_{un}$ slowly tends to this asymptotical value as the correlation dimension increases in chaotic processes or the correlations become weaker in stochastic ones.

#### 2.3.2. Global Clustering Coefficient

Another commonly studied topological property in networks is the global clustering coefficient C, which was introduced by Watts and Strogatz [27]. It gives information about to what extent nodes tend to be clustered together. The coefficient definition is based on triplets of nodes. This term refers to groups of three nodes which are connected by two or three edges. In the last situation, if one of these groups reaches the maximum possible number of edges among the three nodes, then it is called a closed triplet. According to this, C is defined as the proportion of closed triplets over the total number of triplets (open and closed) and it can be computed through the adjacency matrix of the network [17]:

(3) $$C=\frac{\sum_{i,j,k}A_{ij}A_{jk}A_{ki}}{\sum_{i}k_{i}\left(k_{i}-1\right)}$$

where $k_{i}$ is the degree of node i and if the denominator is null, then C is set to zero.

Note that this quantity is a unique value for each network and is in the range $\left[0,\;1\right]$. The closer the clustering coefficient is to one, the more clustered the network is.

## 3. Results

### 3.1. Degree Centrality Computation

**Figure 4:**
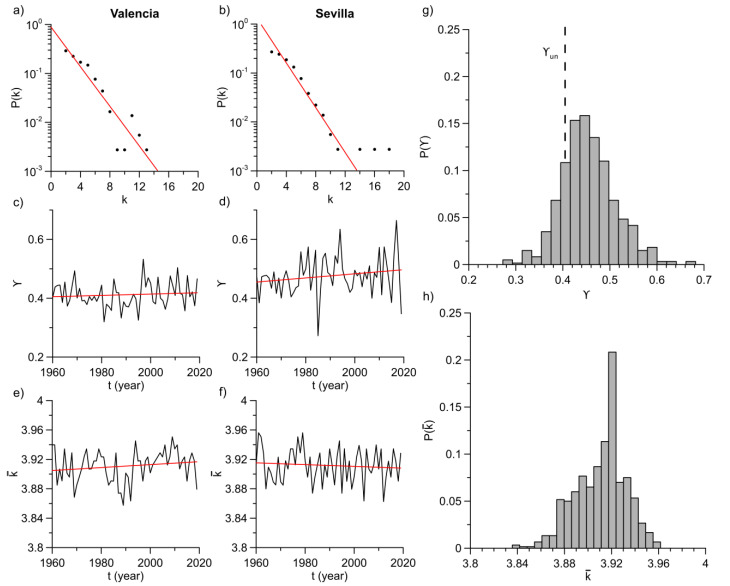
The left margin: (**a**,**b**) Degree distributions of Valencia and Sevilla stations in year 2019, respectively. Red lines are the least-square fits of values. (**c**,**d**) Annual evolution of slopes obtained from the previous linear fits (γ exponent) for Valencia and Sevilla, respectively. (**e**,**f**) Annual evolution of mean values of degree for the same stations. Red lines in every case represent the least-squares fits of curves. The right margin: (**g**) Normalized histogram of γ exponent obtained for all locations and years. Dashed line represents the theoretical value for an uncorrelated random series ($\gamma_{un}=\ln\left(3/2\right)$). (**h**) Normalized histogram of mean degree for all locations and years.

As previously stated, after separating temperature anomalies into annual time series, the HVG algorithm was computed for each case. Next, the degree centrality was obtained from the corresponding networks and the mean values of every network and degree distributions were studied. An example of this can be observed in Figure 4 for Valencia and Sevilla stations.

Figure 4a,b illustrates the degree distributions of the mentioned stations in year 2019 in log-linear plots. It can be appreciated how these distributions well fit to an exponential function of the form: $P\left(k\right)\;\sim \;\exp\left(-\gamma k\right)$. Slopes obtained from least-square fit are different values of the characteristic exponent γ. In a similar way, the rest of stations replicate the expected theoretical behavior of these curves.

Figure 4c,d depict the annual evolution of these exponents for the same stations while Figure 4e,f show annual evolution of average degree. Linear fits were computed in every case and outcomes are displayed in Table 2. The authors investigated *t*-tests of these fits to determine whether these network properties evolve in a similar way to the trends identified in annual means of temperature anomalies (see Figure 3). *t*-tests verify or reject the null hypothesis of that slope is different from zero. For this reason, if the null hypothesis is rejected, the curve exhibits a statistically significant trend. Interestingly, *t*-tests approved null hypothesis at a 95% confidence level for annual average degree in every station and for the γ exponent curves in almost every location (see Table 2). This outcome, together with rather low values of Pearson correlation coefficients, suggest that γ and average degree do not show statistically significant trends. As a consequence, they must oscillate around a mean value. These mean values were also computed, and they are discussed next.

In Table 3, we show the mean values and their corresponding standard errors for γ exponents and average degree in the whole period of 60 years. These values are all quite close to each other despite large distances among stations. In fact, mean degree absolutely coincide in values up to the second decimal with the same error: $\bar{k}=3.91\pm 0.02$, while γ is in the range $\left[0.41,\;0.48\right]$. Mean average degree is associated to the average number of connections that nodes have in networks. This means that a high value of this parameter—and thus, more connected graphs—will be related to irregular time series. In this case, every location time series is smoother, rather than rougher, and this feature remains in time. As $\gamma >\ln\left(3/2\right)$, temperature anomalies are situated in the region of correlated stochastic processes. Nonetheless, they are rather close to the limit of an uncorrelated random process, so correlations are very weak [14]. This also contributes to the smoothness of time series, since correlation tends to decrease the number of nodes with high degree.

When results from each year and station are analyzed together, one can find that their normalized histograms are also centered around one value and take the form of gaussian distributions.

On the one hand, the histogram of γ is depicted on Figure 4g. It is centered around 0.45 with a relatively important amount of values grouped to the right side of the exponent value from a white noise process, $\gamma_{un}$. However, it also displays a significant number of them falling on the left side (approximately 19% of all yearly time series analyzed). These last values are all equally distributed along the years and over different stations. This outcome points to the fact that although this parameter suggests a “mean behavior” that has a stochastic character, it also exhibits a chaotic character in a shorter time scale.

On the other hand, we also illustrate the normalized histogram of average degree on Figure 4h. This histogram shows a sharper distribution with the peak being the previously mentioned result from Table 3, what could explain the coincident results for every location.

In summary, degree distributions are quite similar in average independently of locations, although some differences can be observed. Mean degree is the same for all locations and this can indicate that it might not be affected by local conditions, such as coastal proximity or latitude. In contrast, although γ exponents are close to each other, a distinct strength of correlations can be observed among different locations. Contrary to what Braga et al. found for river flow fluctuations [21], we found an absence of trends. This points to the fact that such parameters can be considered as good constant properties for temperature anomalies, without being affected by any kind of trend from climate change.

The study of the character of nonlinearities in temperature anomalies suggests that signals exhibit an overall behavior which can be classified as stochastic, although in shorter time scale some yearly time series can be classified as chaotic. Lacasa and Toral found that although extrinsic noise was well captured by the HVG algorithm, it failed to discern chaotic from stochastic character for intrinsic noise [14]. More sophisticated methods such as the ϵ entropy and the finite size Lyapunov exponent analysis have also shown some difficulties to distinguish nonlinear nature of signals due to the finiteness of the observational data [28]. Nonetheless, climate system has been often defined as a nonlinear system involving both chaotic and stochastic components [29,30]. It is possible that in shorter time scales our results can be strongly affected by mesoscale convective phenomena—such as Atlantic or Mediterranean (cold drop) depressions landfalls in the Iberian Peninsula—that provide a more chaotic nature to signals.

### 3.2. Clustering Coefficient Computation

**Figure 5:**
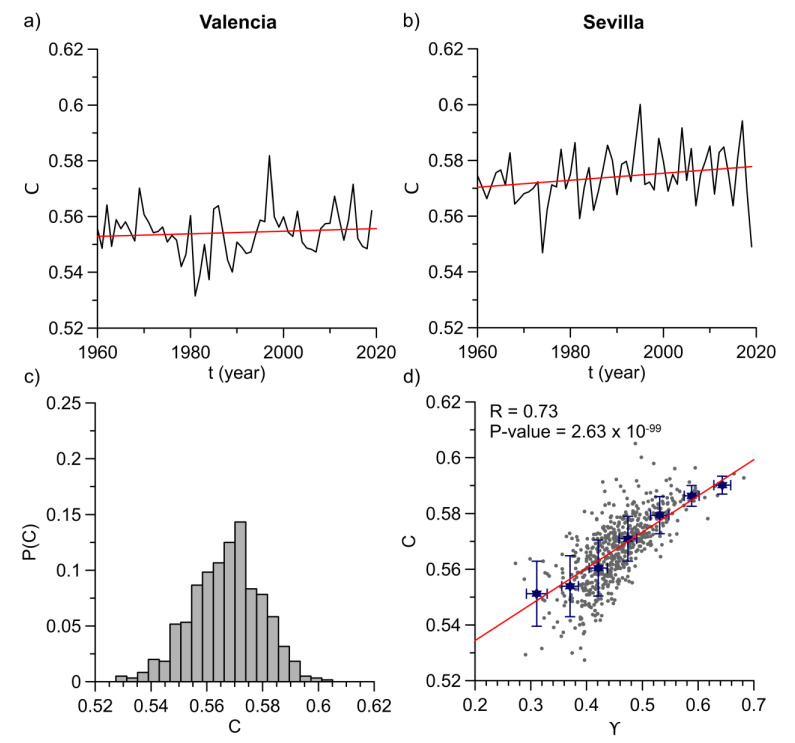
(**a**) Annual evolution of global clustering coefficient (C along the years for Valencia station. Red line represents the least-squares fit of the curve. (**b**) The same plot for Sevilla station. (**c**) Normalized histogram of C for all years and locations. (**d**) Scatter plot (grey dots) of C vs. the characteristic degree exponent (γ). Correlation coefficient and *p*-value for testing the null hypothesis that C and γ are not correlated. This *p*-value is smaller than the 95% significance level (less than 0.05), thus the correlation is statistically significant. Blue stars are window average values obtained from seven bins of equal size in γ axis and error bars are standard deviations. Red line is the least-squares fit of these average values.

The computed global clustering coefficients of HVGs from Valencia and Sevilla stations are depicted vs. time on Figure 5a,b. The rest of locations shows similar behaviors. It can be observed how these plots are analogous to those obtained from the average degree and γ parameters. Again, trends were tested with *t*-tests. It was found that the null hypothesis was accepted in most cases as well as in the degree results leading to no significant trends for the majority of locations (see Table 2). Only three stations had a significant trend at a 95% confident level. Nonetheless, the orders of magnitude in slopes are too low and their respective Pearson correlation coefficients are no more than 0.10. Therefore, the global clustering coefficient agrees with the previous analyzed topological properties and yearly values can be considered as oscillations around a mean. This result suggests that there is no annual evolution in complexity of time series structure. Moreover, linear trends of anomalies do not affect it.

Mean values of annual clustering coefficients and their standard errors are displayed on Table 3 for every location. As it can be seen in the table, they are all really close to each other, varying from 0.55 to 0.57, although standard errors are lower than in the case of γ. This shows that the obtained networks exhibit a complex structure where nodes have some tendency to be clustered. It also suggests a quite similar behavior in different stations which, as it was commented before, remain almost constant along the years. For illustrative purposes, Figure 5c shows the normalized histogram obtained from every year and location. This confirms that the complex structure is rather similar in every case with a higher or lower degree of clustering. These results also take the form of a gaussian distribution.

Finally, authors compared all results to check whether some kind of relationship could exist among the three parameters (average degree, degree exponent and clustering coefficient). A statistically significant correlation between clustering coefficient and γ exponent was found. This correlation can be observed on Figure 5d. Grey dots represent the relation between both distributions with a high correlation coefficient of 0.73 and extremely low *p*-value for the correlation test at a 95% confidence level (2.63 × 10^−99^). This last outcome is much less than the significance level, which means that we can certainly reject the null hypothesis that C and γ are not correlated. In the same figure, it can also be seen the window average over seven bins of equal size in γ axis. They fit to a straight line with a Pearson coefficient of 0.99 and a slope of 0.13 ± 0.01. Braga et al. also got a coupling on average between these two topological properties when they studied river flow fluctuations in Brazil [21]. In that work, an exponential function was the best fit to the average values. Temperature anomalies, conversely, exhibit a linear coupling of degree exponent and global clustering coefficient. This also corroborates that both properties behave in a similar way.

## 4. Conclusions

Daily mean temperature anomalies show common HVGs structures over different locations which also remain almost constant in a relative long period of time (up to 60 years). Three studied topological parameters (average degree, degree exponent and global clustering coefficient) do not show statistically significant annually trends in most cases, although annual mean values of anomalies do show them. Indeed, these anomalies are clearly affected by a positive trend which can be related to the global conditions of rising temperatures in the context of climate change, but this fact apparently does not affect the topological properties of networks.

When mean values were analyzed, they showed a coincident mean average degree and similar degree exponents and clustering coefficients in every location. According to this, these properties are more related to the natural process itself than to the local variations in climate or geographical conditions. In fact, one can clearly notice that HVG algorithm characterizes the nature of the time series, as explained below. Firstly, mean degree invariance points to a similar smoothness in every time series, which is characteristic of dissipative processes where the air temperature is involved. Secondly, similar degree exponents are higher than the theoretical value for an uncorrelated process. This fact suggests that the underlining process is mainly stochastic with weak correlations. Lastly, clustering coefficients—which are related to the tendency of nodes to be clustered together—also give some information about these correlations. This last consideration is corroborated since it is found a great correlation between degree exponents and clustering coefficients which can be well fitted to a line.

Finally, the characteristics of these constant properties could be useful to expand databases for climate models validation. However, some problems remain open for future studies, such as the confirmation of these results on more locations governed by other climate conditions, or the appearance of different new outcomes. Other open research fields for future works include the application of other variants from the VG framework or HVGs together with Shannon–Fisher plane method [31]. This last methodology could be studied in future works on real data.

## Figures and Tables

**Table 1:** Meteorological stations name, coordinates and altitude.

Station Name	Short Name	Latitude (°N)	Longitude (°W)	Altitude (m)
Albacete air base	Albacete	38.95	1.86	702
Badajoz airport	Badajoz	38.88	6.81	185
Barcelona airport	Barcelona	41.29	−2.07	4
Bilbao airport	Bilbao	43.30	2.91	42
Burgos airport	Burgos	42.36	3.62	891
La Coruña	La Coruña	43.37	8.42	58
Málaga airport	Málaga	36.67	4.48	5
Sevilla airport	Sevilla	37.42	5.88	34
Valencia	Valencia	39.48	0.37	11
Zaragoza airport	Zaragoza	41.66	1.00	249

**Table 2:** Slopes with standard errors and Pearson correlation coefficients of linear fits of mean degree (k), characteristic exponent of degree distribution (γ) and global clustering coefficient (C) for each location over time. Values in bold refer to statistically significant trends at a 95% confidence level given by *t*-tests.

Station	$m_{k}$ $\left(\times 10^{-4}\right)$	$\sigma_{m_{k}}$ $\left(\times 10^{-4}\right)$	$R_{k}$	$m_{\gamma }$ $\left(\times 10^{-4}\right)$	$\sigma_{m_{\gamma }}$ $\left(\times 10^{-4}\right)$	$R_{\gamma }$	$m_{C}$ $\left(\times 10^{-4}\right)$	$\sigma_{m_{C}}$ $\left(\times 10^{-4}\right)$	$R_{C}$
Albacete	2.2	1.7	0.03	−1.9	4.3	0.00	0.6	0.7	0.01
Badajoz	−0.2	1.7	0.00	7.1	4.2	0.05	1.0	0.7	0.04
Barcelona	−2.4	1.4	0.05	0.7	3.2	0.00	**1.7**	**0.8**	**0.06**
Bilbao	−1.4	1.7	0.01	**7.3**	**3.5**	**0.07**	**1.9**	**0.7**	**0.10**
Burgos	1.7	1.4	0.02	3.6	4.3	0.01	0.8	0.7	0.02
La Coruña	1.7	1.6	0.02	6.4	4.3	0.04	1.1	0.8	0.03
Málaga	0.9	1.4	0.01	−4.7	2.8	0.05	−0.2	0.7	0.00
Sevilla	−1.2	1.7	0.01	7.0	4.8	0.04	1.3	0.7	0.05
Valencia	2.0	1.6	0.03	2.3	3.2	0.01	0.5	0.6	0.01
Zaragoza	−1.8	1.6	0.02	7.4	5.2	0.03	**2.0**	**0.8**	**0.10**

**Table 3:** Average values and standard errors of mean degree (k), characteristic exponent of degree distribution (γ) and global clustering coefficient (C) for the 60-year period in each location.

Station	$\mu_{k}$	$\sigma_{k}$	$\mu_{\gamma }$	$\sigma_{\gamma }$	$\mu_{C}$	$\sigma_{C}$
Albacete	3.91	0.02	0.46	0.06	0.57	0.01
Badajoz	3.91	0.02	0.47	0.06	0.57	0.01
Barcelona	3.91	0.02	0.43	0.04	0.56	0.01
Bilbao	3.91	0.02	0.46	0.05	0.57	0.01
Burgos	3.91	0.02	0.48	0.06	0.57	0.01
La Coruña	3.91	0.02	0.46	0.06	0.57	0.01
Málaga	3.91	0.02	0.43	0.04	0.56	0.01
Sevilla	3.91	0.02	0.48	0.07	0.57	0.01
Valencia	3.91	0.02	0.41	0.04	0.55	0.01
Zaragoza	3.91	0.02	0.47	0.07	0.57	0.01

## Data Availability

Publicly available datasets were analyzed in this study. This data can be found here: http://www.aemet.es/es/datos_abiertos/AEMET_OpenData.
